# The impact of pseudophakia on vision-related quality of life in the general population – The Gutenberg Health Study

**DOI:** 10.18632/aging.101208

**Published:** 2017-03-28

**Authors:** Alexander K. Schuster, Norbert Pfeiffer, Andreas Schulz, Stefan Nickels, René Höhn, Philipp S. Wild, Maria Blettner, Thomas Münzel, Manfred E. Beutel, Karl J. Lackner, Urs Vossmerbaeumer

**Affiliations:** ^1^ Department of Ophthalmology, Mainz University Medical Center, Mainz, Germany; ^2^ Preventive Cardiology and Preventive Medicine, Center for Cardiology, University Medical Center of the Johannes Gutenberg-University Mainz, Mainz, Germany; ^3^ Department of Ophthalmology, Inselspital, University Hospital of Bern, Bern, Switzerland; ^4^ Center for Thrombosis and Hemostasis, University Medical Center of the Johannes Gutenberg-University Mainz, Mainz, Germany; ^5^ DZHK (German Center for Cardiovascular Research), partner site Rhine-Main, Mainz, Germany; ^6^ Department of Biomedical Statistics, University Medical Center of the Johannes Gutenberg-University Mainz, Mainz, Germany; ^7^ Center for Cardiology, University Medical Center Mainz, Mainz, Germany; ^8^ Department of Psychosomatic Medicine and Psychotherapy, University Medical Center Mainz, Mainz, Germany; ^9^ Institute of Clinical Chemistry and Laboratory Medicine, University Medical Center Mainz, Mainz, Germany

**Keywords:** quality of life, epidemiology, cataract surgery, pseudophakia, aphakia

## Abstract

Cataract surgery is the most frequently performed surgical procedure worldwide. We aim to determine the prevalence of having implanted an artificial lens (pseudophakia) and of no lens (aphakia) and to compare visual function.

As part of the Gutenberg Health study, a population-based cross-sectional study was conducted in Germany. An ophthalmological examination including slit-lamp examination was conducted. Prevalence including 95% confidential intervals were calculated and analyses were conducted for systemic and ocular associated factors with pseudophakia using multivariable logistic regression models. Vision-related quality of life was assessed using a standardized questionnaire and Rasch transformation.

14,696 people were included. Of these, 1.55% [1.36%–1.77%] had unilateral pseudophakia and 3.08% [2.81%-3.37%] had bilateral pseudophakia. Unilateral aphakia was present in 21 people and bilateral aphakia in 2 people. Pseudophakia was independently associated with age, higher body weight and lower body height, diabetes and smoking. Vision-related quality of life values were similar for those with bilateral phakia and pseudophakia but were lower for those with unilateral pseudophakia.

The pseudophakia status is related to several cardiovascular risk factors, indicating a relationship to an aging effect that causes premature lens opacification. Bilateral pseudophakia can almost imitate the physiological condition of phakia except for the need to use glasses.

## INTRODUCTION

Cataract has been identified as the leading cause of visual impairment and blindness worldwide [1]. Cataract surgery is the most frequently performed surgical procedure worldwide. The opacified lens is removed and either replaced with an artificial intraocular lens (pseudophakia), or the resulting refractive error is corrected with spectacles or with contact lenses in cases of aphakia.

While the frequency and outcome of cataract surgery in developing countries is well investigated [2-10], the prevalence of pseudophakia or aphakia in high income countries is less well investigated at present [11-14]. During the last two decades, several technological improvements have made cataract surgery a relatively safe and common procedure. Even clear lens extractions are performed during refractive surgery [15].

In the Gutenberg Health Study we had the opportunity to investigate the prevalence of pseudophakia and aphakia in Germany and to evaluate associated factors. Furthermore, we investigated vision-related quality of life and tested the hypothesis whether vision-related quality of life in persons with pseudophakia is different compared to bilateral phakic people.

## RESULTS

A total of 14,696 study subjects were included in this analysis. Detailed descriptions of the study sample characteristics are provided in Table 1. Data for 314 people (2%) were missing.

**Table 1:** Characteristics of the baseline sample of the German population-based Gutenberg Health Study (GHS), 2007-2012, stratified according to eye status (bilateral phakic persons, unilateral pseudophakia and bilateral pseudophakia)

Subjects:	Bilateral phakic persons (13993)	Unilateral pseudophakia (228)	Bilateral pseudophakia (452)
Sex (Women)	49.4% (6918)	51.3% (117)	52.4% (237)
Age [y]	54.0 (45.0/64.0)	68.0 (63.0/72.0)	70.0 (65.0/72.0)
SES (median and interquartile-range)	13.00 (9.00/17.00)	11.00 (8.00/15.00)	10.00 (8.00/13.33)
Residence (rural)	46.5% (6506)	52.2% (119)	48.9% (221)
**Cardiovascular** **Risk factors**:			
Diabetes (yes) ^1^	8.7% (1216)	17.1% (39)	24.5% (110)
Obesity (yes) ^2^	24.7% (3452)	29.4% (67)	39.2% (177)
Smoking (yes)	19.8% (2762)	9.7% (22)	14.0% (63)
Hypertension (yes) ^3^	48.6% (6797)	64.5% (147)	72.1% (326)
Dyslipidemia (yes) ^4^	34.1% (4761)	43.2% (98)	47.9% (216)
FH of MI/Stroke (yes) ^5^	22.0% (3076)	22.4% (51)	25.4% (115)
**Anthropometry**:			
BMI [kg/m^2^]	27.3±5.0	28.1±4.7	29.1±5.2
Weight [kg]	79.7±16.6	78.5±15.4	81.0±15.8
Height [cm]	171±9	167±10	167±9
Waist [cm]	94.3±13.9	97.3±13.4	100.0±13.8
Hip [cm]	102.0±9.9	102.6±9.1	104.1±10.5
WHR	0.924±0.092	0.947±0.090	0.960±0.088
**Lipids**:			
Cholesterol [mmol/l]	221±41	224±38	220±44
HDL [mg/dl]	57.3±15.6	60.0±17.4	57.3±16.7
LDL [mg/dl]	139±36	140±33	138±37
Triglycerides [mg/dl]	105.0 (78.0/147.0)	104.2 (79.2/140.0)	114.0 (83.0/159.0)
**Ophthalmic parameters**:		**Pseudophakic/** **phakic eyes**	
Glasses (yes)	88.8% (12431)	96.5% (220)	97.8% (442)
Distance glasses (yes)	67.2% (9402)	81.6% (186)	68.8% (311)
Contact lenses (yes)	4.7% (651)	1.3% (3)	0% (0)
Reading glasses (yes)	71.8% (10049)	95.2% (217)	92.9% (420)
Intraocular pressure [mmHg]	14.3±2.8	14.0±2.8	14.0±3.3
Central corneal thickness [μm]	554±35	555±35	553±37
Refraction: Sphere [dpt]	−0.123±2.433	−0.63±1.38 / -0.28±2.83	−0.500±1.165
Cylinder [dpt] in median (interquartile-range)	−0.375 (−0.750/-0.125)	−0.75 (−1.21/-0.50) / −0.50 (−1.00/-0.25)	−0.625 (−1.000/-0.375)
Visual acuity [logMAR]	0.07±0.12	0.24±0.28/ 0.22±0.27	0.16±0.17
Cataract as determined by slit-lamp examination	31.1% (4348)	70.6% (161)	Not applicable
**Eye diseases (self-reported)**:			
Glaucoma (yes)	2.0% (275)	10.5% (24)	7.3% (33)
AMD (yes)	0.4% (56)	2.2% (5)	1.5% (7)
Corneal disease (yes)	1.9% (263)	4.8% (11)	4.0% (18)

Means ± standard deviation are reported for normally distributed data, absolute and relative frequency are reported for dichotomous variables, and medians and quartile ranges are reported otherwise. Abbreviations: (AMD: age-related macular degeneration; BMI: body mass index; FH: family history; HDL: high-density lipoproteins; LDL: low-density lipoproteins; MI: myocardial infarction; SES: socio-economic status; WHR: waist-hip ratio; ^1^ HBA_1C_ ≥ 6.5 or diabetic medication use or diagnosis by physician; ^2^ BMI ≥ 30 kg/m^2^; ^3^ hypertension diagnosed by a physician or systolic blood pressure ≥ 140 mmHg or diastolic blood pressure ≥ 90 mmHg or anti-hypertensive medication;^4^ LDL/HDL ratio >3.5 or lipid-lowering medication use; ^5^ Family history of myocardial infarction or family history of stroke.)

### Prevalence of pseudophakia and aphakia

**Figure 1:**
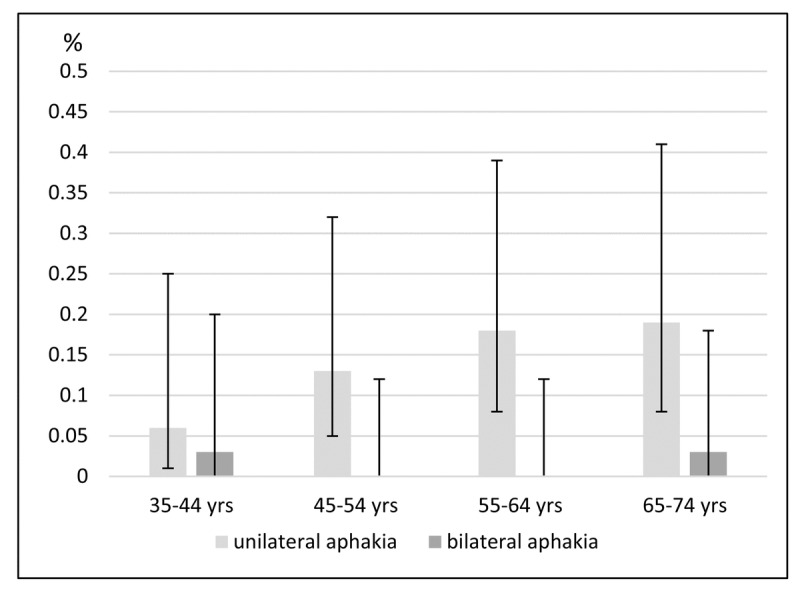
Age-specific prevalence and 95% confidence intervals for aphakia (in percentages) in 35- to 74-year-old people in Germany (the Gutenberg Health Study)

Two hundred twenty-eight people of the study population (1.55%; 95% confidence interval [1.36%-1.77%]) had unilateral pseudophakia, while 452 (3.08% [2.81%-3.37%]) had bilateral pseudophakia. Unilateral aphakia was present in 21 people (0.14% [0.09%-0.22%]), and bilateral aphakia was present in 2 people (0.01% [0.00%-0.05%]). The prevalence data for each decade of age is given in Figure 1 showing no age dependency.

**Figure 2:**
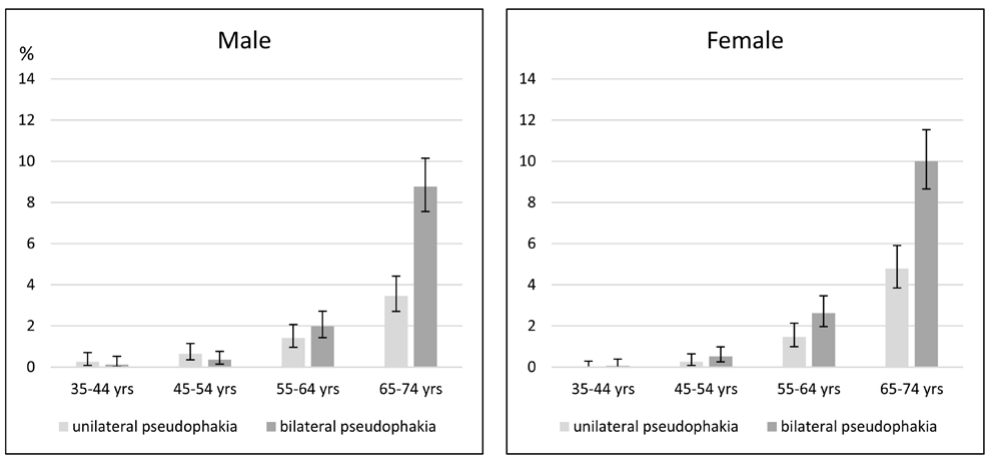
Age- and sex-specific prevalence and 95% confidence intervals for pseudophakia (in percentages) in 35- to 74-year-old people in Germany (the Gutenberg Health Study)

The weighted prevalence data for Germany for people aged 35 to 74 years are 1.36% [1.18% - 1.57%] for unilateral pseudophakia and 2.64% [2.39% - 2.91%] for bilateral pseudophakia. Unilateral aphakia was present in 0.14% [0.09% - 0.21%], and bilateral aphakia was present in 0.01% [0.00% - 0.05%]. The prevalence data are described separately for each decade of age and for each sex in Figure 2.

### Reasons for aphakia

The reasons for unilateral aphakia were eye injury (11 people), unilateral congenital cataract (1 person), high myopia (1 person), recurrent uveitis (1 person) and unknown (7 people). Bilateral congenital cataract was the cause of bilateral aphakia in one person, while the other person had a perforating injury in one eye and later developed a complicated retinal detachment in the other eye.

Of the 228 people with unilateral pseudophakia, one had an iris-fixated anterior chamber intraocular lens following phakic lens removal because of an eye trauma, and one person had a chamber-angle-supported intraocular lens following unilateral cataract surgery in childhood.

Of the 452 people with bilateral pseudophakia, one had a secondary implantation of an iris-fixated anterior chamber intraocular lens after endophthalmitis, and one had an iris-fixated anterior chamber intraocular lens in addition to a posterior chamber intraocular lens.

### Characteristics of pseudophakic persons

Logistic regression analyses showed that higher age, higher body weight, smaller body height, diabetes and smoking were independently associated with presence of any pseudophakia (unilateral or bilateral; Table 2).

**Table 2:** Analysis of anthropometric and cardiovascular associations with pseudophakia versus phakia as the lens status using a logistic regression model in 35- to 74-year-old people in Germany (the Gutenberg Health Study)

	Any pseudophakia vs. bilateral phakic persons (14380 persons)				Unilateral pseudophakia vs. bilateral phakic persons (13938 persons)				Bilateral pseudophakia vs. bilateral phakic persons (14157 persons)			
	OR	Lower 95%CI	Upper 95%CI	p-value	OR	Lower 95%CI	Upper 95%CI	p-value	OR	Lower 95%CI	Upper 95%CI	p-value
Sex (Women)	1.05	0.82	1.35	0.70	0.83	0.55	1.26	0.38	1.19	0.773	2.117	0.27
Age [10y]	4.51	3.96	5.15	**< 0.0001**	3.43	2.77	4.19	**< 0.0001**	5.36	4.53	6.39	**0.00032**
SES	1.01	0.99	1.03	0.33	1.03	1.00	1.06	0.09	1.00	0.98	1.02	0.97
Weight [kg]	1.01	1.00	1.02	**0.005**	1.01	0.99	1.02	0.43	1.01	1.00	1.02	**0.0023**
Height [cm]	0.98	0.97	0.99	**0.005**	0.97	0.95	1.00	**0.028**	0.98	0.96	1.00	**0.032**
Diabetes	1.40	1.12	1.74	**0.003**	1.16	0.77	1.71	0.47	1.50	1.16	1.94	**0.0021**
Smoking	1.30	1.01	1.70	**0.039**	0.85	0.52	1.33	0.50	1.60	1.19	2.13	**0.0016**
HDL [mg/dl]	1.00	1.00	1.00	0.63	1.01	1.00	1.02	0.25	1.00	0.99	1.01	0.85
LDL [mg/dl]	1.00	1.00	1.00	0.36	1.00	1.00	1.00	0.72	1.00	1.00	1.00	0.42
Triglycerides [mg/dl]	1.00	1.00	1.00	0.052	1.00	1.00	1.00	0.13	1.00	1.00	1.00	0.15

Abbreviations: OR: odds ratio; 95%CI: 95% confidence interval; SES: socio-economic status.

For unilateral pseudophakia, higher age and lower body height were independently associated, while for bilateral pseudophakia, higher age and smoking status were independently associated (Table 2).

Self-reported eye diseases were more common in people with unilateral pseudophakia compared with those with bilateral pseudophakia or phakic persons.

Among those with unilateral pseudophakia, 10.5% reported having glaucoma, compared with 7.3% of people with bilateral pseudophakia and 2.0% of phakic people. While the presence of age-related macular degeneration was similar in all three groups, corneal diseases were more common in people with unilateral pseudophakia (Table 1). Cataracts were found on slit-lamp examination in 31% of the bilateral phakic participants, while 71% of those with unilateral pseudophakia had a cataract in the other eye

### Visual function and vision-related quality of life

Distance-corrected visual acuity differed between the three groups: phakic people showed the best mean visual acuity (logMAR: 0.07±0.12), followed by those with bilateral pseudophakia (logMAR: 0.16±0.17) and those with unilateral pseudophakia (phakic eyes: 0.22±0.27, pseudophakic eyes: 0.24±0.28).

Vision-related quality of life data, in terms of Rasch transformed scales of the NEI VFQ-25 values, are provided in Table 3. The people with bilateral phakia had the highest scores for socioemotional scale and visual function scale. followed by those with bilateral pseudophakia, while the people with unilateral pseudophakia had the lowest scores.

**Table 3:** Descriptive of vision-related quality of life measured by the NEI VFQ-25 questionnaire in persons with phakic lenses or pseudophakia in 35-74 year old persons in Germany (the Gutenberg Health Study)

Mean±SD	Bilateral phakic persons (13993)	Unilateral pseudophakia (228)	Bilateral pseudophakia (452)
Long form visual function scale (LFVFS)	86.8±10.6	78.6±15.3	84.1±12.1
Long form socioemotional scale (LFSES)	96.2±7.3	90.3±14.1	93.5±9.8

Visual function scale and socioemotional scale in long form was calculated using Rasch analysis.

After the age- and sex-standardization of vision-related quality of life in people aged 60 years and more, the quality of life of those with bilateral pseudophakia was comparable to that of the people with bilateral phakia in the visual function scale, while the people with unilateral pseudophakia had reduced vision-related quality of life measurements (Table 4).

**Table 4:** Age- and sex-standardized vision-related quality of life measured by the NEI VFQ-25 questionnaire in persons with phakic lenses or pseudophakia in persons above 60 years in Germany (the Gutenberg Health Study)

Mean±SD	Bilateral phakic persons	Unilateral pseudophakia	Bilateral pseudophakia
Long form visual function scale (LFVFS)	85.1±11.0	79.3±15.4	84.9±11.6
Long form socioemotional scale (LFSES)	95.1±8.3	90.9±14.3	93.8±9.5

Visual function scale and socioemotional scale in long form was calculated using Rasch analysis.

### Using glasses

The use of glasses for distance vision was higher in the unilateral pseudophakic group (82%) than in the bilateral groups (pseudophakic: 69%; phakic 67%). For reading purpose, 72% of the bilateral phakic persons stated using glasses, compared to 95% of those with unilateral and 93% of those with bilateral pseudophakia. When incorporating age and sex differences, 79% of bilateral phakic persons and only 69% of persons with bilateral pseudophakia reported using glasses for distance vision (both values refer to people over 60 years old). Glasses for reading were used in similar proportions (97% of bilateral phakic people, 96% of those with unilateral pseudophakia and 93% of those with bilateral pseudophakia).

### Intra-individual comparison

When we analyzed the characteristics of the pseudophakic versus the phakic eye of the same participants (228 people), we found greater astigmatism in the pseudophakic eye (median −0.50 diopters (25%-/75%-quartile −1.00/-0.25) versus −0.75 diopters (−1.21/-0.50), p-value 0.008), while spherical refraction (−0.28 +/− 2.83 vs. −0.63 +/− 1.38 diopters) and distance-corrected visual acuity (phakic eyes: 0.22±0.27, pseudophakic eyes: 0.24±0.28 in logMAR) did not differ.

## DISCUSSION

For the first time, we report the prevalence of pseudophakia and aphakia in Germany in a population based study and investigate the causes of aphakia after the introduction of phacoemulsification and the implantation of a posterior-chamber intraocular lens. We found that 1.55% of the 35- to 74-year-old population has unilateral pseudophakia, and approximately 3% has bilateral pseudophakia. These people are more likely to be older, to suffer from diabetes and to smoke. Sex is not associated with pseudophakia status.

### Prevalence of pseudophakia and associated factors

The prevalence of pseudophakia in our cohort is similar to that reported in other regions of the world: e.g., the Singapore Malay Eye Study (4.7%) [4], the Beaver Dam Eye Study in Wisconsin (3.1%) [16], the Australian Blue Mountains Eye Study (6.0%) [17], the Barbados Eye Study (3.0%) [18] and the Central India Eye and Medical Study (5.0%) [7]; however, other Indian studies of urban regions have reported figures as high as 9.4% [9,19]. In China, the prevalence of pseudophakia is lower, as reported in the Beijing Eye Study 2006 [20] and 2009 [5] and the China Nine-Province Survey [10]. Because these prevalence data are not standardized to the same age and sex distribution, they are not directly comparable. In addition, most of the studies did not distinguish between unilateral and bilateral pseudophakia. When comparing changes in lens extractions over time, the Beaver Dam Eye Study found a remarkable 6.5-fold increased incidence between 1990 and 2010 [21].

As in the Central India Eye and Medical Study and the Tanjong Pagar Survey from Singapore [22], diabetes mellitus was independently associated with having had cataract surgery. It is not clear whether diabetic subjects were referred to an ophthalmologist earlier to screen for diabetic retinopathy. If so, decreased vision would have been detected earlier; however, it is more likely that diabetes is a risk factor for cataract [23] and therefore, diabetic people are more likely to have had cataract surgery. Nevertheless, cataract and diabetic retinopathy is both linked to an aging organism. Similarly, the presence of a pseudophakic intraocular lens was related to smoking status, which is a known risk factor for cataract formation [24].

In the multivariable analysis in the present study, pseudophakia was independently associated with a heavier body weight and a shorter body height. A recent meta-analysis of cohort studies showed that age-related cataract is associated with obesity [25], while a lower body height was associated with cataract in the Physicians' Health Study [26]. Socioeconomic status was independent of the presence of pseudophakia, which is not surprising for a country with a health insurance system that covers almost 100 percent of the population and reimburses the costs of cataract surgery with intraocular lens implantation.

The standardized prevalence of aphakia is lower in the German population than reported for other study populations in developed countries or regions [5,11,27]. The cause of aphakia in our study population was primarily trauma and aphakia in Germany can be considered as individual cases.

### Visual acuity and vision-related quality of life

Visual function differed between the participants with bilateral phakia, unilateral pseudophakia and bilateral pseudophakia. Distance-corrected visual acuity was normal in the bilateral phakic participants and was almost normal in the subjects with bilateral pseudophakia. In subjects with unilateral pseudophakia, visual acuity did not differ between the phakic and the pseudophakic eye but was lower compared with phakic subjects or with subjects having bilateral pseudophakia.

Vision-related quality of life was highest in bilateral phakic participants in our study cohort, followed by the bilateral pseudophakic group and the unilateral pseudophakic group. When incorporating age- and sex-standardization, the bilateral phakic and pseudophakic groups were comparable: the unidimensional score “visual function scale” was almost identical. This indicates that bilateral pseudophakia permits a visual quality of life that is very similar to the physiological condition. Our finding of lower vision-related quality of life in subjects with unilateral pseudophakia than in those with bilateral pseudophakia is in agreement with previous work by Javitt et al. [28]. Javitt et al. reported that patients who underwent surgery in both eyes had greater improvement in vision-related quality of life than did those who underwent surgery in only one eye. They concluded that there seems to be a beneficial restoration of binocular vision when both eyes are treated. Similarly, To et al. showed that the increase in vision-related quality of life after bilateral cataract surgery is related to an improved stereopsis and contrast sensitivity and not to visual acuity [29]. In addition, brightness and color perception increases when replacing an opacified lens with a clear implant. The causes for lower visual acuity and lower vision-related quality of life in peoples with unilateral pseudophakia might be the higher proportion of eye diseases in these people in our study: we found an increase in self-reported glaucoma and corneal diseases among those with unilateral pseudophakia compared with those with bilateral pseudophakia and with bilateral phakic persons. Similar results were found for the presence of cataract: over 70% of subjects with unilateral pseudophakia had a cataract in the other eye.

### Using glasses

As suspected, cataract surgery with intraocular lens implantation leads to a need for glasses when monofocal intraocular lenses are implanted, as is the standard treatment covered by health insurance in Germany. In our study cohort, 98% of the study participants over the age of 60 years reported wearing glasses either for distance or near vision. Further analysis showed that bilateral pseudophakia is linked to less need to wear glasses for distance vision (69% for bilateral pseudophakia vs. 79% for bilateral phakia vs. 82% for unilateral pseudophakia), while the proportions of persons using reading glasses were comparable between each lens status group. However, it is to be noted that the primary goal of cataract surgery is replacing the opacified crystalline lens by a clear implant, not to correct refractive errors.

When comparing the pseudophakic eye with the phakic eye in the same person, we did detect a higher rate of astigmatism in the pseudophakic eye (0.25 diopters) but no difference in sphere. Cataract surgery may introduce the degree of corneal astigmatism [30].

There are methodological limitations to the study: first, the pseudophakic and aphakic status was determined by slit-lamp examination using an undilated pupil. Because most of our study participants were Caucasians, our conclusions should be considered valid for this ethnicity only and cannot be generally applied to people from other ethnic and genetic backgrounds. In addition, the age range of our study population was 35 to 74 years; therefore, the prevalence data are only valid for this age range and there may be a further increase of prevalence towards the very old persons. Response to study invitation has to be considered as limitation in most population-based studies. In our study, the response was 60% [31]. As we do not have any knowledge about eye health in non-responders, we are not able to adjust for that people with low vision might be less likely to participate. Regarding differences in response within our study population, persons with an age between 35 and 44 years showed a lower, but similar response for men and women [32]. As prevalence of pseudophakia is very low before the age of 50 years due to the rare condition of early cataract development, this will rather not have influenced our results. In addition, women over the age of 65 years did less likely participate in our study than did men in this age or younger persons. As we found a comparable increase of prevalence of pseudophakia between men and women towards older age, the smaller response of older women is rather unlikely to have distorted our results.

In summary, the prevalence of pseudophakia ranges from 0.2% in the 35- to 44-year age group to 13.4% in the 65- to 74-year age group in Germany. The pseudophakia status is related to several cardiovascular risk factors, indicating a relationship with aging mechanisms that caused premature lens opacification that was treated with cataract surgery. Vision-related quality of life measurements indicate that bilateral pseudophakia can almost imitate the physiological condition of phakia except for the need to use glasses. Aphakia is currently a rare condition in Germany and is primarily caused by trauma or the consequence of congenital cataract.

## METHODS

The Gutenberg Health Study (GHS) is a prospective, population-based, observational cohort study conducted in the Rhine-Main region in Germany. Our sample of 15,010 participants was randomly drawn from local governmental registry offices. The cohort was equally stratified by sex, urban and rural residence within each decade of age. More details regarding the study design are described in Höhn et al. [31]. For each participant, a comprehensive ophthalmological examination was conducted. Objective refraction (Humphrey Automated Refractor/Keratometer (HARK) 599, Carl Zeiss Meditec AG, Jena, Germany) and distance-corrected visual acuity, non-contact tonometry (Nidek NT-2000, Nidek Co, Japan), slit-lamp examination of the anterior segment and funduscopy was performed, and ophthalmic conditions (e.g., phakia, pseudophakia and aphakia) were documented using standardized documentation sheets. These variables (aphakia, pseudophakia) were validated using refraction and the ophthalmic medical history.

Venous blood was collected during the fasting state (i.e., overnight fasting if the subject was examined before 12 p.m. and 5-hour fasting if the subject was examined after 12 p.m.). Diabetes mellitus was determined in individuals with HbA1c ≥6.5%, those who were taking diabetic medication and those who had been diagnosed by a physician.

The subjects' socioeconomic status (SES) was defined according to the SES index used for the German Health Update 2009 (GEDA) and ranged from 3 to 21.[33]

Vision-related quality of life was assessed using the German version of the National Eye Institute 25-Item Visual Function Questionnaire (NEI VFQ-25) [34,35]. The questionnaire was self-administered as print-out. The data were extracted by study staff using double-entry to ensure data quality. The NEI VFQ-25 consists of 25 questions that are originally used to calculate twelve subscores and one global vision-related quality of life (QoL) score, with values ranging from 0=worst to 100=best. Previous studies showed that these scores underlie multidimensionality [36-38].

We therefore chose a Rasch-based analyse, as conducted by several studies [39-41]. Rasch analysis enables to transform the raw questionnaire data into an interval-level scale. We utilized the transformation algorithm as suggested by Pesudovs et al. [39]. The polarity of several items was adapted to ensure that a higher score corresponds to lower performance. The answer option “Stopped doing this for other reasons or not interested in doing this” was set to missing. The filter question 15 (“Are you currently driving, at least once in a while?”) and the related questions 15a and 15b were excluded. The tables provided by Pesudovs et al. [39] were utilized to assign for each question the raw measures to Rasch-transformed scores on person-level. Based on these measures, the visual function scale (long form: LFVFS) and the socioemotional scale (long form: LFSES), were calculated [39]. For the LFVFS, the Rasch-transformed scores of questions 2, 5, 6, 7, 8, 9, 10, and 14 were add up and transformed to a scale of 0 to 100, where 0 corresponds to worst performance, and that 100 corresponds to the sum of all items answered with the least reduction in performance. Analogue, the scores of LFSES (questions 11, 13, 17, 18, 20, 21, 22, 23, 24, 25) have been calculated.

### Informed consent

The study protocol and study documents were approved by the local ethics committee of the Medical Chamber of Rhineland-Palatinate, Germany (reference no. 837.020.07; original vote: 22.3.2007, latest update: 20.10.2015). According to the tenets of the Declaration of Helsinki, written informed consent was obtained from all participants prior to entering them in the study.

### Study sample

This study comprised the baseline examination of the GHS and included subjects who were aged 35 to 74 years at the time of examination. The examinations took place between April 2007 and March 2011. There was no exclusion criterion.

### Data and statistical analysis

All primary and secondary variables were first tested for normal distribution. Medians, interquartile ranges, minimums and maximums were calculated for all primary and secondary variables. For variables that were normally distributed, means and standard deviations were computed as well.

First, age-specific prevalence of pseudophakia and aphakia were calculated. To estimate the overall standardized prevalence, the age/sex-distribution of the the German population from the year 2014 was used [42]. Associated anthropometric and cardiovascular factors were evaluated using multivariable logistic regression models. Models comparing #1 people with any pseudophakia to people without lens replacement, #2 people with unilateral pseudophakia (and phakia in the fellow eye) to those with bilateral phakia (i.e., without lens replacement), #3 people with bilateral pseudophakia to people with bilateral phakia were analyzed. The included covariables were sex, age (as continuous variable), SES, body weight, body height, diabetes, smoking and concentration of lipids (high-density lipoproteins, low-density lipoproteins, triglycerides).

An intra-individual comparison (pseudophakic versus phakic eye) using a paired t-test was performed to evaluate differences in refraction (sphere, astigmatism) and visual acuity.

Vision-related quality of life were analyzed descriptively. Age- and sex-standardization of vision-related quality of life and wearing spectacles in people aged of 60 years and more was performed to compare these variables between people with unilateral pseudophakia, bilateral pseudophakia and bilateral phakia.

The data were processed using statistical analysis software (R version 3.1.1 [2014-07-10]).

This study was performed as an explorative study to analyze the prevalence of pseudophakia and aphakia and the factors associated with these conditions. All p-values should be regarded as continuous parameters that reflect the level of evidence and are therefore reported exactly.

## References

[1] Congdon NG, Friedman DS, Lietman T (2003). Important causes of visual impairment in the world today. JAMA.

[2] Bourne RR, Dineen BP, Ali SM, Huq DM, Johnson GJ (2003). Outcomes of cataract surgery in Bangladesh: results from a population based nationwide survey. Br J Ophthalmol.

[3] Dandona L, Dandona R, Naduvilath TJ, McCarty CA, Mandal P, Srinivas M, Nanda A, Rao GN (1999). Population-based assessment of the outcome of cataract surgery in an urban population in southern India. Am J Ophthalmol.

[4] Lavanya R, Wong TY, Aung T, Tan DT, Saw SM, Tay WT, Wang JJ, SiMES team (2009). Prevalence of cataract surgery and post-surgical visual outcomes in an urban Asian population: the Singapore Malay Eye Study. Br J Ophthalmol.

[5] Liu B, Xu L, Wang YX, Jonas JB (2009). Prevalence of cataract surgery and postoperative visual outcome in Greater Beijing: the Beijing Eye Study. Ophthalmology.

[6] Murthy GV, Gupta S, Ellwein LB, Munoz SR, Bachani D, Dada VK (2001). A population-based eye survey of older adults in a rural district of Rajasthan: I. Central vision impairment, blindness, and cataract surgery. Ophthalmology.

[7] Nangia V, Jonas JB, Gupta R, Khare A, Sinha A (2011). Prevalence of cataract surgery and postoperative visual outcome in rural central India Central India Eye and Medical Study. J Cataract Refract Surg.

[8] Salomão SR, Soares FS, Berezovsky A, Araújo-Filho A, Mitsuhiro MR, Watanabe SE, Carvalho AV, Pokharel GP, Belfort R, Ellwein LB (2009). Prevalence and outcomes of cataract surgery in Brazil: the São Paulo eye study. Am J Ophthalmol.

[9] Vashist P, Talwar B, Gogoi M, Maraini G, Camparini M, Ravindran RD, Murthy GV, Fitzpatrick KE, John N, Chakravarthy U, Ravilla TD, Fletcher AE (2011). Prevalence of cataract in an older population in India: the India study of age-related eye disease. Ophthalmology.

[10] Zhao J, Ellwein LB, Cui H, Ge J, Guan H, Lv J, Ma X, Yin J, Yin ZQ, Yuan Y, Liu H (2010). Prevalence and outcomes of cataract surgery in rural China the China nine-province survey. Ophthalmology.

[11] Barañano AE, Wu J, Mazhar K, Azen SP, Varma R, Los Angeles Latino Eye Study Group (2008). Visual acuity outcomes after cataract extraction in adult latinos. The Los Angeles Latino Eye Study. Ophthalmology.

[12] Congdon N, Vingerling JR, Klein BE, West S, Friedman DS, Kempen J, O'Colmain B, Wu SY, Taylor HR, Eye Diseases Prevalence Research Group (2004). Prevalence of cataract and pseudophakia/aphakia among adults in the United States. Arch Ophthalmol.

[13] Daien V, Le Pape A, Heve D, Carriere I, Villain M (2015). Incidence and Characteristics of Cataract Surgery in France from 2009 to 2012: A National Population Study. Ophthalmology.

[14] Solborg Bjerrum S, Mikkelsen KL, la Cour M (2015). Epidemiology of 411 140 cataract operations performed in public hospitals and private hospitals/clinics in Denmark between 2004 and 2012. Acta Ophthalmol.

[15] Fernández-Vega L, Alfonso JF, Villacampa T (2003). Clear lens extraction for the correction of high myopia. Ophthalmology.

[16] Klein BE, Klein R, Linton KL (1992). Prevalence of age-related lens opacities in a population. The Beaver Dam Eye Study. Ophthalmology.

[17] Mitchell P, Cumming RG, Attebo K, Panchapakesan J (1997). Prevalence of cataract in Australia: the Blue Mountains eye study. Ophthalmology.

[18] Leske MC, Connell AM, Wu SY, Hyman L, Schachat A (1997). Prevalence of lens opacities in the Barbados Eye Study. Arch Ophthalmol.

[19] Nirmalan PK, Thulasiraj RD, Maneksha V, Rahmathullah R, Ramakrishnan R, Padmavathi A, Munoz SR, Ellwein LB (2002). A population based eye survey of older adults in Tirunelveli district of south India: blindness, cataract surgery, and visual outcomes. Br J Ophthalmol.

[20] Xu L, Cui T, Zhang S, Sun B, Zheng Y, Hu A, Li J, Ma K, Jonas JB (2006). Prevalence and risk factors of lens opacities in urban and rural Chinese in Beijing. Ophthalmology.

[21] Klein BE, Howard KP, Lee KE, Klein R (2014). Changing incidence of lens extraction over 20 years: the Beaver Dam eye study. Ophthalmology.

[22] Foster PJ, Wong TY, Machin D, Johnson GJ, Seah SK (2003). Risk factors for nuclear, cortical and posterior subcapsular cataracts in the Chinese population of Singapore: the Tanjong Pagar Survey. Br J Ophthalmol.

[23] Prokofyeva E, Wegener A, Zrenner E (2013). Cataract prevalence and prevention in Europe: a literature review. Acta Ophthalmol.

[24] Panday M, George R, Asokan R, Ve Ramesh S, Velumuri L, Choudhari NS, Boddupalli SD, Sunil GT, Vijaya L (2016). Six-year incidence of visually significant age-related cataract: the Chennai eye disease incidence study. Clin Experiment Ophthalmol.

[25] Pan CW, Lin Y (2014). Overweight, obesity, and age-related cataract: a meta-analysis. Optom Vis Sci.

[26] Schaumberg DA, Glynn RJ, Christen WG, Hankinson SE, Hennekens CH (2000). Relations of body fat distribution and height with cataract in men. Am J Clin Nutr.

[27] Lau J, Michon JJ, Chan WS, Ellwein LB (2002). Visual acuity and quality of life outcomes in cataract surgery patients in Hong Kong. Br J Ophthalmol.

[28] Javitt JC, Brenner MH, Curbow B, Legro MW, Street DA (1993). Outcomes of cataract surgery. Improvement in visual acuity and subjective visual function after surgery in the first, second, and both eyes. Arch Ophthalmol.

[29] To KG, Meuleners LB, Fraser ML, Do DV, Duong DV, Huynh VA, To QG, Phi TD, Tran HH, Nguyen ND (2014). The impact of cataract surgery on vision-related quality of life for bilateral cataract patients in Ho Chi Minh City, Vietnam: a prospective study. Health Qual Life Outcomes.

[30] Masket S, Wang L, Belani S (2009). Induced astigmatism with 2.2- and 3.0-mm coaxial phacoemulsification incisions. J Refract Surg.

[31] Höhn R, Kottler U, Peto T, Blettner M, Münzel T, Blankenberg S, Lackner KJ, Beutel M, Wild PS, Pfeiffer N (2015). The ophthalmic branch of the Gutenberg Health Study: study design, cohort profile and self-reported diseases. PLoS One.

[32] Wild PS (2011). Analysis of Baseline Recruitment and Non-Response in the Population-Based Gutenberg Health Study. Johannes Gutenberg-University Mainz.

[33] Lampert T, Kroll LE, Müters S, Stolzenberg H (2013). [Measurement of the socioeconomic status within the German Health Update 2009 (GEDA)]. Bundesgesundheitsblatt Gesundheitsforschung Gesundheitsschutz.

[34] Mangione CM, Lee PP, Gutierrez PR, Spritzer K, Berry S, Hays RD, National Eye Institute Visual Function Questionnaire Field Test Investigators (2001). Development of the 25-item National Eye Institute Visual Function Questionnaire. Arch Ophthalmol.

[35] Franke GH, Esser J, Voigtländer A, Der Mähner N (1998). National Eye Institute Visual Function Questionnaire (NEI-VFQ)–Erste Ergebnisse zur psychometrischen Überprüfung eines Verfahrens zur Erfassung der Lebensqualität bei Sehbeeinträchtigten. Z Med Psychol.

[36] Labiris G, Katsanos A, Fanariotis M, Tsirouki T, Pefkianaki M, Chatzoulis D, Tsironi E (2008). Psychometric properties of the Greek version of the NEI-VFQ 25. BMC Ophthalmol.

[37] Kovac B, Vukosavljevic M, Djokic Kovac J, Resan M, Trajkovic G, Jankovic J, Smiljanic M, Grgurevic A (2015). Validation and cross-cultural adaptation of the National Eye Institute Visual Function Questionnaire (NEI VFQ-25) in Serbian patients. Health Qual Life Outcomes.

[38] Mollazadegan K, Huang J, Khadka J, Wang Q, Yang F, Gao R, Pesudovs K (2014). Cross-cultural validation of the National Eye Institute Visual Function Questionnaire. J Cataract Refract Surg.

[39] Pesudovs K, Gothwal VK, Wright T, Lamoureux EL (2010). Remediating serious flaws in the National Eye Institute Visual Function Questionnaire. J Cataract Refract Surg.

[40] Dougherty BE, Bullimore MA (2010). Comparison of scoring approaches for the NEI VFQ-25 in low vision. Optom Vis Sci.

[41] Petrillo J, Cano SJ, McLeod LD, Coon CD (2015). Using classical test theory, item response theory, and Rasch measurement theory to evaluate patient-reported outcome measures: a comparison of worked examples. Value Health.

[42] Statistisches Bundesamt. GENESIS-Online Datenbank (2016). Ergebnisse auf Grundlage des Zensus 2011.

